# Yizhiqingxin Formula Alleviates Cognitive Deficits and Enhances Autophagy *via* mTOR Signaling Pathway Modulation in Early Onset Alzheimer’s Disease Mice

**DOI:** 10.3389/fphar.2019.01041

**Published:** 2019-09-17

**Authors:** Yang Yang, Zhiyong Wang, Yu Cao, Jiangang Liu, Peng Li, Hao Li, Meixia Liu

**Affiliations:** ^1^Institute of Geriatrics, Xiyuan Hospital, China Academy of Chinese Medical Sciences, Beijing, China; ^2^Beijing Key Laboratory of Pharmacology of Chinese Materia Region, Institute of Basic Medical Sciences of Xiyuan Hospital, China Academy of Chinese Medical Sciences, Beijing, China

**Keywords:** Alzheimer’s disease, Yizhiqingxin formula, autophagy, mTOR, APP/PS1 mice

## Abstract

Alzheimer’s disease (AD) is the most common type of dementia worldwide. The deposition of amyloid β (Aβ) is one of the most important pathological changes in AD. Autophagy, which mediates degradation of toxic proteins and maintains normal neuronal function, is dysfunctional in AD; dysfunctional autophagy is believed to be a critical pathological feature of AD. Here, we evaluated the *in vitro* and *in vivo* effects of a traditional Chinese medicinal formula called Yizhiqingxin formula (YQF) on autophagy. We determined that treatment with a high dose of YQF improved spatial memory and decreased the hippocampal Aβ burden in *APP/PS1* mice, an early onset AD model. Transmission electron microscopy and immunohistochemical data revealed that YQF enhanced autophagosome formation and also increased the levels of LC3II/LC3I and Beclin1. Further, we found that YQF treatment promoted autophagic activity by inhibiting the phosphorylation of the Mammalian target of rapamycin (mTOR) at the Ser2448 site. Moreover, the level of 4EBP1 increased after YQF intervention, indicating a suppression of mTOR signaling. YQF was also found to promote autophagosome degradation, as indicated by the decreased p62 levels and increased cathepsin D and V-ATPase levels. Taken together, YQF could improve spatial learning in *APP*/*PS1* mice and ameliorate the accumulation of Aβ while promoting autophagy *via* mTOR pathway modulation.

## Introduction

Alzheimer’s disease (AD), manifested by core clinical features of progressive memory loss and executive dysfunction, is the most common type of dementia worldwide. In China, the current prevalence of AD in people over 65 is 3.21% (Jia et al., 2014). The aggravating trend of aging population also drives the increasing number of AD patients. According to the World Alzheimer Report 2018, it is estimated that there are about 47 million Alzheimer’s patients worldwide, with 10 million new cases each year (Alzheimer’s Association, 2018). As the disease progresses, most patients eventually need constant care and help with most basic daily activities, exacting a heavy burden on families and society (Jia et al., 2018). AD therapy is reported to be the most expensive treatment in the U.S. (Souchet et al., 2018).

Autophagy is an evolutionarily conserved intracellular process responsible for the lysosomal degradation and removal of degraded and aggregated proteins, and damaged intracellular organelles to maintain cellular homeostasis (Ryter et al., 2013). This robust quality control mechanism is critical in neurons to support their long-term viability and functionality. Multiple studies have identified defective autophagic activity in several neurodegenerative diseases, especially in AD, which is characterized by amyloid β (Aβ) peptide aggregation. Moreover, both *in vitro* and *in vivo* studies have shown that autophagy retards neuronal degeneration and age-associated cognitive disorders by the clearance of accumulated toxic proteins. An abnormal alteration of autophagy and Aβ deposition has been observed in AD patients, along with a hyperactivation of the mammalian target of rapamycin (mTOR) pathway (Sun et al., 2014; Tramutola et al., 2017). Consistent with these clinical findings, overwhelming data suggest a vicious cycle between Aβ deposition and mTOR hyperactivity (Oddo, 2012; Talboom et al., 2015).

Yizhiqingxin formula (YQF; previously called Fuzheng Quxie Decoction **Supplementary Data Sheet 1**) is a traditional Chinese formula composed of *Panax ginseng* (radix), *Coptis chinensis* (rhizome), and *Conioselinum anthriscoides ‘Chuanxiong’* (rhizome). Our previous work found that YQF could alleviate cognitive deficits and tau hyperphosphorylation in SAMP8 mice (Yang et al., 2017); vascular endothelial growth factor (VEGF) and VEGF receptor were the other molecular targets of YQF (Wang et al., 2018). However, the effects of YQF on autophagy and the underlying mechanisms are not clear. In the present study, we investigate the potential roles of YQF on autophagy and mTOR signaling in *APP/PS1* mice and *APP/PS1* double transgenic HEK293 cells. Data revealed that YQF could ameliorate cognitive deficits and decrease Aβ deposition, suggesting that YQF is a potential candidate for the treatment of AD.

## Materials and Methods

### Animals

Sixty 3-month-old male *APP*/*PS1* transgenic mice were randomly divided into five groups: Model group, Rapa group (2.24 mg·kg^−1^·d^−1^), YQF high dose group (YQF-H, 10.4 g·kg^−1^·d^−1^ raw drugs), YQF medium dose group (YQF-M, 5.2 g·kg^−1^·d^−1^ raw drugs), and YQF low dose group (YQF-L, 2.6 g·kg^−1^·d^−1^ raw drugs). Twelve male C57BL/6 mice with the same genetic background at 3 months of age were used as control. Each group was given 24 weeks of gavage intervention. Drugs used in Rapa and YQF-L groups were equivalent doses suggested in the clinic. The Control and Model groups were given equal volumes of distilled water. The other groups were given corresponding drugs. Mice were all housed in groups of three to four per cage and maintained in a specific pathogen-free conditions at 22 ± 2°C with automatic light cycles (12 h light/dark) and relative humidity of 50–70%. Animals received food and water *ad libitum* and were allowed to adapt to the environment for 1 week before experiment. All experiments were carried out in accordance with the guidelines for animal care and were approved by the animal ethics committee of the Ethics Committee of Xiyuan Hospital, China Academy of Chinese Medical Sciences (NO. 2018XLC004-1).

### Reagents

Rabbit monoclonal anti-beta amyloid 1–42 (ab201060) antibody and rabbit polyclonal anti-LC3 II (ab48394), anti-p62 (ab91526), anti-mTOR (ab2732), and anti-cathepsin D (ab75852) antibodies were procured from Abcam (UK). Rabbit polyclonal anti-ATPase (sc-28801) antibody was procured from Santa Cruz (USA). Rabbit polyclonal anti-p-mTOR (5536S), anti-4EBP1 (9644S), anti-P70S6K (2708S), anti-Beclin 1 (3738S), and anti-LAMP1 (3243S) antibodies were procured from Cell Signaling Technology (USA). Mouse monoclonal anti-GAPDH (YM3029) antibody was purchased from Immunoway (USA). Mouse monoclonal anti-*β-actin* (sc-69879) antibody was purchased from Santa Cruz (USA). Aβ ELISA kit (DAB142) was purchased from RD (USA).

### Drug Preparation

YQF is composed of *Panax ginseng* (radix), *Coptis chinensis* (rhizome), and *Conioselinum anthriscoides ‘Chuanxiong’* (rhizome), with a weight ratio of 9:5:6. Raw drugs were purchased from Beijing Kangrentang Pharmaceutical Co. Ltd. They were deposited at the Herbarium of National Resource Center for Chinese Materia Medica (CMMI), China Academy of Chinese Medical Sciences. The codes for *Panax ginseng* (radix), *Coptis chinensis* (rhizome), and *Conioselinum anthriscoides ‘Chuanxiong’* (rhizome) specimens were 0220582LY0015, 420528LY0075, and 331127LY0805, respectively. The extraction was done by the Department of Pharmaceutics of Xiyuan Hospital, China Academy of Chinese Medical Sciences. Per gram extraction was obtained from 3.71 grams raw drug mixture. Seven major bioactive compounds (gginsenoside Rg1, ginsenoside Re, ginsenoside Rb1, coptisine, berberine, ligustilide, and ferulic acid) were identified using HPLC (high performance liquid chromatography) (Yang et al., 2017).

### Cell Culture, Gene Transfection, and Drug Intervention Protocols

The human embryonic kidney cell line (HEK293 cells) was purchased from National Infrastructure of Cell Line Resource (NO: 3111C0001CCC000128). Cells were cultured in Dulbecco’s modified Eagle medium (DMEM; Hyclone, USA) supplemented with penicillin–streptomycin (100 IU/ml and 100 *µ*g/ml, Gibco, USA) and 10% fetal bovine serum (Gibco, USA) in a humidified atmosphere of 5% CO_2_ at 37°C. Cells were passaged using 0.25% trypsin with 0.02% EDTA every 3–4 days. During transfection, HEK293 cells were cultured in Opti-MEM I Serum Medium (Gibco, USA) and transfected with APP695 plasmid (Addgene, USA) with Lipofectamine 2000 (Invitrogen, USA) following manufacturers’ instructions. The *APP695* gene HEK293 (APP-HEK293) cell clones were selected using puromycin (4 μg/ml, Amresco, USA). PSEN1dE9 plasmids (Beijing Borui Technology Co., Ltd., China) were transfected into the APP-HEK293 cells [clones were selected using G418 (1 mg/ml, Amresco, USA)], and then the *APP/PS1* double gene transfected HEK293 cells stably expressing *APP695* and *PSEN1dE9* genes (*APP*/*PS1* double transgenic HEK293 cells) were obtained.

After gene transfection, HEK293 cells were divided into the Control group (treated with Control group serum), and *APP*/*PS1* double transgenic HEK293 cells were divided into the Model group, YQF-H group, YQF-M group, and YQF-L group and were treated with serum containing YQF.

### Cell Viability Assay

Cell viabilities were measured by the Cell Counting Kit-8/WST-8 kit (CCK8, Dojindo, Japan) following the manufacturer’s instruction. Briefly, cells were plated in a 96-well plate at a density of 1 *×* 10^5^/ml. CCK8 mixed with DMEM without phenol red at a ratio of 1:9 was added to the 96-well plate, and then the plate was incubated in an incubator for 1–2 h. Optical density readings were measured with a Microplate Reader (Bio Tek, USA) at 450 nm wavelength.

### Preparation of Serum Containing YQF (YQF-Serum)

Forty 3-month-old male Sprague Dawley rats purchased from Huafukang Biological Technologies [Beijing, China, NO: SCXK (Beijing) 2014-0004] were divided into four groups: YQF high dose group (YQF-H, 18 g·kg^−1^·d^−1^, herbal medicine), YQF medium dose group (YQF-M, 9 g·kg^−1^·d^−1^, herbal medicine), YQF low dose group (YQF-L, 1.8 g·kg^−1^·d^−1^, herbal medicine), and Control group (control, equal volume of distilled water) with 10 rats in each group. Rats in the four groups received a daily gavage with the respective treatment for 1 week. Subsequently, the rats were anaesthetized by intraperitoneal injection with pentobarbital sodium. Blood was drawn from the abdominal aorta and centrifuged at 3000 rpm for 10 min. The YQF-Serum so obtained was used for further experiments.

### Morris Water Maze Tests

Spatial memory was assessed using the Morris water maze (MWM) test, as described previously (Yang et al., 2017). Briefly, a tank (diameter 120 cm, height 50 cm, painted white) filled with water (22°C) to the depth of 40 cm was used. The water was stained with nontoxic white dye. A white platform (diameter, 10 cm; height, 15 cm) was placed in the center of the target quadrant 1 cm beneath the water. The place navigation trial consisted of five training days and four trials per day with a 20-min inter-trial interval. Mice were placed into the water from four different starting positions facing the wall, and each trial lasted for 60 s. If a mouse did not reach the platform within 60 s, it was guided to the platform where it had to remain for 10 s. The swimming distance and escape latencies were recorded. One day after the task acquisition (day 5), a probe trial was performed in order to assess the spatial memory. The platform was removed. Animals were put into the tank from the position opposite to the platform and allowed to swim freely for 60 s. The percentage of total time spent in each quadrant and the number of crossings where the platform had been previously located were recorded.

### Analysis of Blood and Brain Tissue for Active Components of YQF

After the MWM experiments, blood samples were collected from rats being transcardially perfused with ice-cold physiological saline for brain tissue isolation. Brains were quickly removed on ice; cerebral cortices were then separated and homogenized in ice-cold physiological saline (2 ml saline per 1 g cortex). Plasma was isolated from the blood samples of each group. Subsequently, liquid chromatography tandem mass spectrometry (LC-MS/MS) was used to detect the active components of YQF present in the blood plasma and brain tissue homogenate.

### Transmission Electron Microscopy Analysis

Cells from *in vitro* experiments and hippocampal tissues obtained from the sacrificed mice, post MWM tests, were fixed with 2.5% glutaraldehyde and washed three times in phosphate-buffered saline (PBS) (pH 7.2), following which they were post fixed in 1% osmium tetroxide, dehydrated in graded ethanol and acetone solution, and embedded in epoxy resin. Sectioning and staining were subsequently performed under general electron microscopy supervision. Autophagosomes in cells of different samples were observed and imaged under a transmission electron microscope at 4000× magnification.

### Immunohistochemistry Assays

After the behavior test, five mice from each group were anesthetized with pentobarbital sodium (30 mg/kg) and were perfused by intracardiac puncture with PBS (4°C) and 4% paraformaldehyde. Immunohistochemistry staining was conducted on 20 mm thick coronal sections as previously reported (Wang et al., 2010). Briefly, the sections were dewaxed, rehydrated, and incubated in PBS containing 3% H_2_O_2_ for 10 min. After washing with tris-buffered saline (TBS), sections were boiled in citric acid buffer for 5 min in a microwave oven and then incubated overnight at 4°C with rabbit antibody against Aβ_1-42_ antibody (1:1000 dilutions). After rinsing, sections were incubated with biotinylated secondary antibody for 1 h, followed by streptavidin peroxidase for 1 h at room temperature. Subsequently, the sections were visualized with 3, 3-diaminobenzidine and analyzed using an inverted microscope (OLYMPUS CKX41, Tokyo, Japan).

To assess the Aβ burden in the cortex and hippocampus, five sections with the same reference position were selected from each mouse (n = 5). The percentage of the total area of Aβ-positive areas compared with the total area of the hippocampus was quantified (in square micrometers). The data were analyzed with the image analysis system (Image-Pro Plus 6.0).

### ELISA

Cell cultures were treated with YQF-serum at the indicated concentrations for the specified durations. The conditioned medium was then collected and subjected to a sandwich ELISA for the Aβ_42_ levels according to the manufacturer’s guidelines.

### Western Blot Analysis

The hippocampus was lysed on ice in lysis buffer and then centrifuged at 12,000g for 10 min at 4°C. The protein content was determined using a BCA protein assay kit (Pierce, Rockford, IL). Proteins (40 mg) were separated by SDS-PAGE electrophoresis and then transferred to 0.45 μm polyvinylidene difluoride membranes (Millipore, Bedford, MA). The membranes were blocked for 2 h in 5% BCA and were then incubated overnight at 4°C with the primary antibody (1:1000 dilutions) followed by a horseradish peroxidase-conjugated secondary antibody (1:2000 dilutions). The blots of proteins of interest were visualized using an ECL advanced Western blotting detection kit (Thermo Fisher Scientific, MA, USA). Quantitative analyses of the protein bands were performed with a Molecular Imager Carestream MI SE system (Rochester, NY, USA).

### Statistical Analysis

Data are expressed as mean ± SEM and were analyzed by GraphPad Prism 7.0 (GraphPad Software, San Diego, CA). MWM latencies were analyzed using two-way analysis of variance (ANOVA) with repeated measures. All the other data were analyzed using one-way ANOVA. Fisher’s least significant difference *post hoc* test was used to test the differences between two groups. Significance level was set at *p* < 0.05.

## Results

### Analysis of Blood and Brain Tissue for Active Components of YQF

Our previous HPLC study revealed that the important active components of YQF are ginsenoside Rg1, ginsenoside Re, ginsenoside Rb1, coptisine, berberine, ligustilide, and ferulic acid (Yang et al., 2017). Here, the LC-MS/MS analyses of active components of YQF in the plasma and brain tissue (Table 1 and **Supplementary DataTable 1**) showed that many alkaloids like berberine, palmatine, worenine, and protopine, and ginsenosides like Rg1, Re, Rb1, Rd, and Rc were found in plasma, while berberine, palmatine, epiberberine, coptisine, and ginsenoside Rb1 were found in brain tissue and ferulic acid of *Conioselinum anthriscoides ‘Chuanxiong’* (rhizome) was found in the plasma but not brain tissue. Since many of the active components of *Conioselinum anthriscoides ‘Chuanxiong’* (rhizome) are volatile, we believe that not all of its components were detected by this method.

**Table 1:** Active components of YQF in the serum and brain tissue of SD rats.

Sample name	Content (ng/mL)				
	Serum-L	Serum-M	Serum-H	Brain-H	Brain-M
Berberine	5.79	6.79	19.3	14.2	6.62
Palmatine	1.37	2.28	1.77	7.07	1.55
Worenine	0.818	0.923	2.18	1.91	ND
Protopine	ND	0.947	1.2	3.69	ND
Epiberberine	1.79	2.42	35.2	8.12	1.99
Coptisine	6.13	8.56	29.6	16.7	4.33
Jatrorrhizine	10.9	ND	19.6	13.4	3.85
Dehydrocorydaline	ND	ND	ND	9.73	ND
Tetrahydrojatrorrhizine	ND	ND	ND	9.12	1.12
Ginsenoside Rg1	ND	ND	22.7	ND	ND
Ginsenoside Re	ND	ND	18.8	ND	ND
Ginsenoside Rb1	87.9	232	514	5.89	ND
Ginsenoside Rd	ND	21.1	48.9	ND	ND
Ginsenoside Rc	31.7	78.9	147	ND	ND
Ferulic acid	1.71	3.96	113	ND	ND

ND, no detected. n = 3. Serum and brains were obtained from SD rats treated with low, medium, or high dose of YQF, and named as Serum-L, Serum-M, Serum-H, Brain-M, and Brain-H, respectively.

### YQF Treatment Alleviates Spatial Memory Deficits in *APP/PS1* Mice

**Figure 1:**
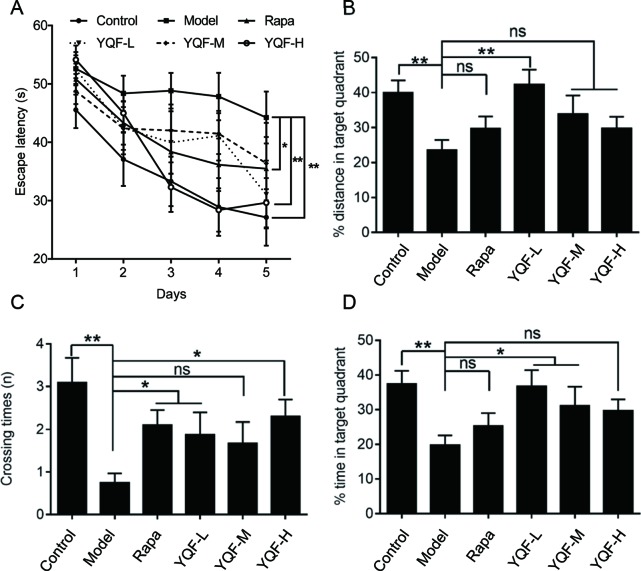
YQF alleviates spatial memory deficits of APP/PS1 mice **(A)** Escape latency, **(B)** Distance in the target quadrant, **(C)** Crossing times, **(D)** Time spent in the target quadrant, n = 8–12; *p < 0.05; **p < 0.01; ns, no significant difference.

APP/PS1 mice cognitive–behavioral changes occur at 3 months of age (Garcia-Alloza et al., 2006), APP/PS1 mice have a substantial number of amyloid deposits by 6 months of age. The plaque burden increases progressively with time (Jankowsky et al., 2004). Therefore, APP/PS1 mice have well simulated the main pathological changes of AD and are currently recognized as the international animal model of AD. To investigate whether a long-term YQF treatment could ameliorate spatial learning deficits in transgenic *APP/PS1* mice, MWM tests were performed. As shown in **Figure 1A**, all mice exhibited a significant decrease in escape latency over time. Two-way ANOVA reported a significant treatment effect on the escape latency [*F*(5, 53) = 3.237, *p* = 0.013]. However, *APP/PS1* mice spent more time searching for the hidden platform than controls (*p* < 0.01 model group vs. control group), indicating a significant cognitive decline. After high dose YQF treatment, the escape latency was shortened remarkably (*p* < 0.01 YQF-H group vs. model group). Rapamycin also reduced the mean latency significantly (*p* < 0.05 Rapa group vs. model group) (**Figure 1A**).

Moreover, in the probe trial, *APP/PS1* mice failed to remember the accurate location of the platform, and the crossover times were remarkably less than the Control group [*F*(5, 54) = 3.615 p = 0.007, *p* < 0.01 model group vs. control group]. Administration of rapamycin or high dose YQF significantly mitigated the deficits (*p* < 0.05 Rapa group vs. model group and *p* < 0.05 YQF-H group vs. model group). Besides, the mice treated with medium dose of YQF spent significantly more time in the target quadrant [*F*(5, 52) = 3.359 *p* = 0.011, *p* < 0.05 YQF-M group vs. model group]. The low dose-treated group also showed a higher percentage of the swimming distance and time in the target quadrant than the Model group [*F*(5, 52) = 3.712 p = 0.006, *p* < 0.01 YQF-L group vs. model group and *F*(5, 52) = 3.359 *p* = 0.011, *p* < 0.05 YQF-L group vs. model group]. These results indicate that YQF treatment improves the spatial learning and memory function in *APP/PS1* mice (**Figures 1B–D**).

### YQF Reduces Aβ Levels in the Hippocampus of *APP/PS1* Mice and *APP/PS1* Double Transgenic HEK293 Cells

**Figure 2:**
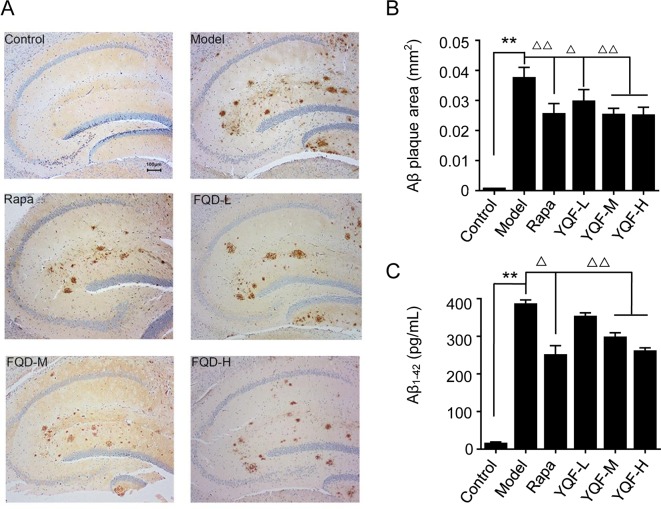
Expression of Aβ_1-42_ in hippocampus of mice **(A** and **B)** and HEK293 cells **(C)**. Data are expressed as mean ± SEM, n = 5, **p < 0.01 compared with the control group, ^△^ *p* < 0.05 and ^△△^ *p* < 0.01 compared with the model group.

To investigate the effect of YQF treatment on Aβ deposition and senile plaque formation, brain slices were stained with a monoclonal antibody against Aβ_1-42_ and quantitative image analysis was performed (**Figures 2A, B**). Results show that in 9-month-old *APP/PS1* mice, immuno-positive diffuse plaques occupied a great deal of the hippocampal areas compared to non-transgenic mice [*F*(5, 24) = 11.618 *p* = 0.000, *p* < 0.01 model group vs. control group]. After YQF treatment, all YQF groups had significantly reduced Aβ burden (*p* < 0.05 YQF-L group vs. model group and *p* < 0.01 YQF-M/H group vs. model group). Rapamycin group also showed a remarkable reduction in Aβ burden (*p* < 0.01 Rapa group vs. model group).

*In vitro* (**Figure 2C**), we also found that *APP/PS1* double transgenic HEK293 cells expressed the highest level of Aβ_1-42_ compared with the other groups [*F*(5,18) = 130.531 p = 0.000, *p* < 0.01 model group vs. control group]; YQF could reduce the expression of Aβ_1-42_, especially in the YQF-M and YQF-H groups (*p* < 0.01 YQF-M/H group vs. model group).

### YQF Promotes the Formation and Degradation of Autophagosome *In Vivo* and *In Vitro*

**Figure 3:**
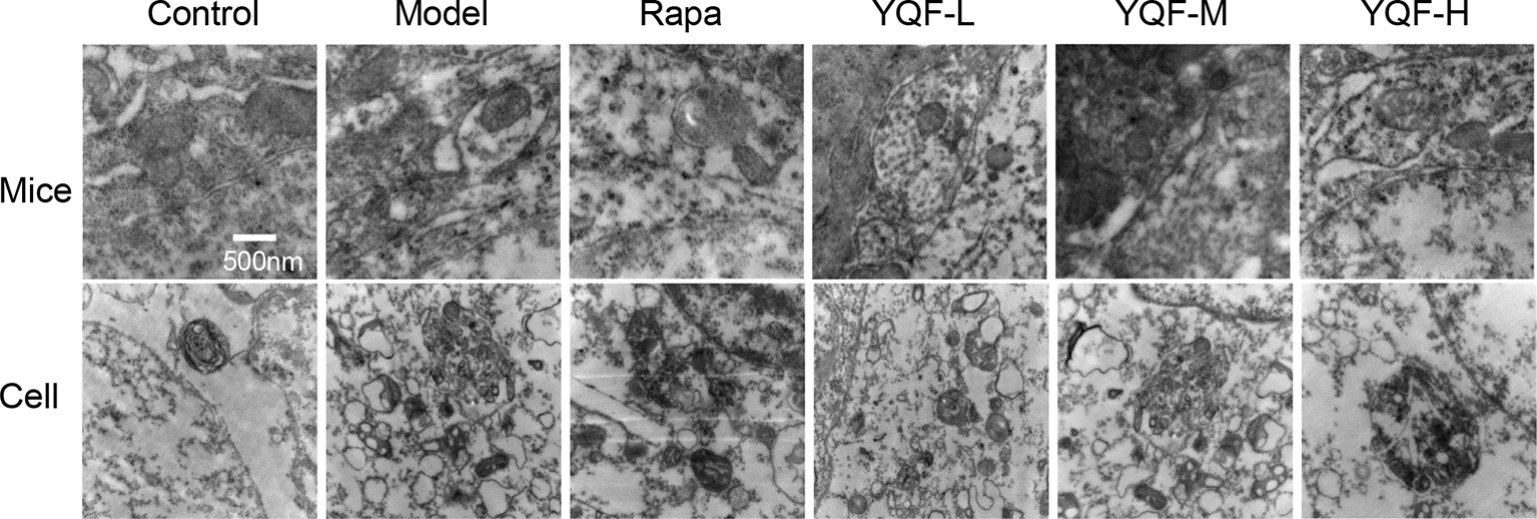
Ultrastructure of hippocampus of 9-month-old APP/PS1 transgenic mice, C57BL/6J mice, HEK293 cells, and APP/PS1 HEK293 cells. Autophagy vesicles were detected by transmission electron microscope after the treatment of rapamycin and YQF (500nm).

By transmission electron microscopy (**Figure 3**), we found using *in vivo* and vitro experiments that many large autophagosomes are assembled in *APP/PS1* mice and cells compared with non-transgenic animals and cells. Mitochondria and other cytoplasmic components could be found in the autophagosomes. Furthermore, rapamycin could assemble large number of autophagosomes in neuronal cells and transgenic HEK293 cells as compared to the Model group. Every cell in the YQF groups exhibited autophagosomes more than that in Model groups but less than in Rapamycin groups. Moreover, we could also find that contents in the autophagic vacuoles of YQF groups were partly degraded, especially in the YQF-H group; apparently, a portion of the autophagic vacuoles were autophagolysosomes.

### YQF Treatment Promotes Autophagy Activity *In Vivo* and *In Vitro*

**Figure 4:**
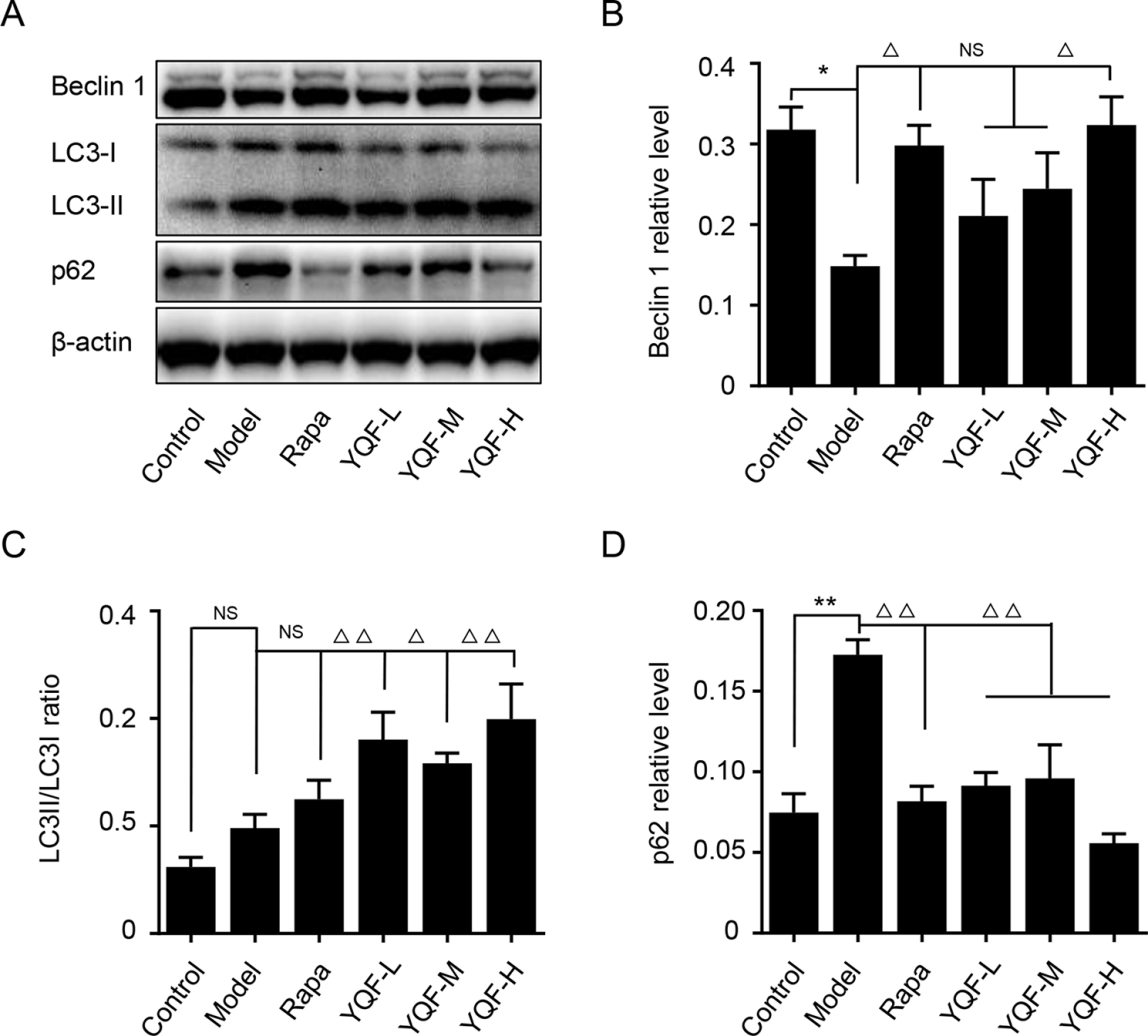
The expression of autophagy related proteins in APP/PS1 and C57BL/6J mice hippocampus intervened with YQF. **(A)** Western blot bands, **(B)** Beclin 1, **(C)** LC3/LC3, **(D)** P62. Data are expressed as mean ± SEM, n = 5, *p < 0.05 and **p < 0.01 compared with the control group, ^△^ *p* < 0.05 and ^△△^ *p* < 0.01 compared with the model group, NS, no significant difference.

**Figure 5:**
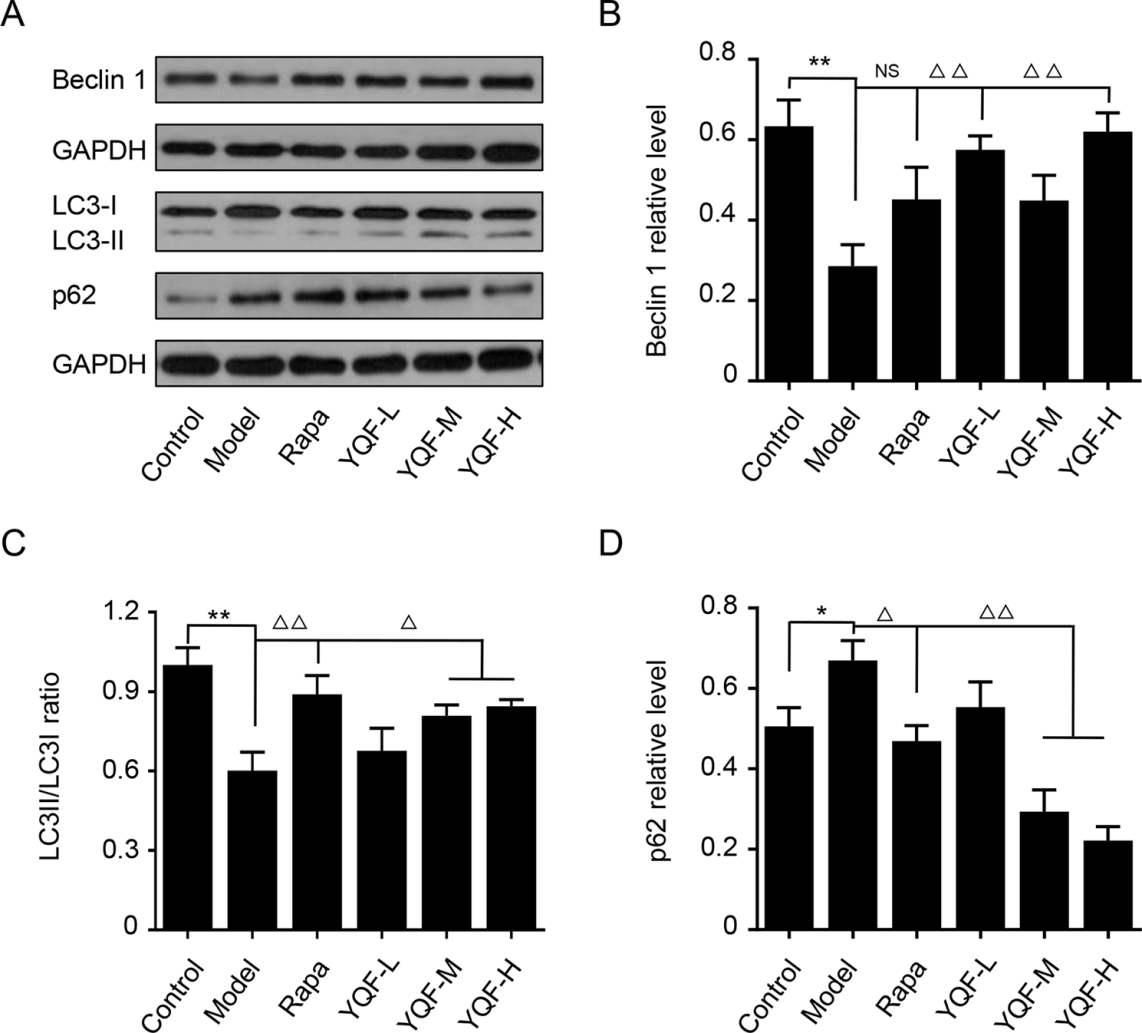
The expression of autophagy related proteins in APP/PS1 and HEk293 cells intervened with YQF. **(A)** Western blot bands, **(B)** Beclin 1, **(C)** LC3II/LC3I, **(D)** P62. Data are expressed as mean ± SEM, n = 3, *p < 0.05 and **p < 0.01 compared with the control group, ^△^*p* < 0.05 and ^△△^*p* < 0.01 compared with the model group, NS, no significant difference.

Beclin 1, LC3, and P62 are protein markers of autophagy activity. *In vitro* and *in vivo* experiments revealed that Beclin 1 levels decreased significantly in Model group mice and cell lines [*χ* *^2^* = 15.565 *p* = 0.008, *p* < 0.05 model group vs. control group *in vivo* and *F*(5,12) = 5.142, *p* = 0.009, *p* < 0.01 model group vs. control group *in vitro*]. With the formation and maturation of the autophagosome, LC3I converts to LC3II, and here, we take the LC3II/LC3I ratio as the sign of the formation of autophagosome. Although we did not find any differences *in vivo* between Model group mice and Control group mice, the LC3II/LC3I ratio of *in vitro* model cell lines decreased significantly [*F*(5,12) = 10.263, *p* = 0.001, *p* < 0.01 model group vs. control group]. Rapamycin could promote the expression of Beclin 1 (*χ* *^2^* = 15.565 *p* = 0.008, *p* < 0.05 Rapa group vs. model group *in vivo*) and LC3II [*F*(5,12) = 10.263, *p* = 0.001, *p* < 0.05 Rapa group vs. model group *in vitro*], similar to the *in vivo* and *in vitro* effects of YQF (Beclin 1 *p* < 0.05 YQF-H group vs. model group *in vivo*, *p* < 0.01 YQF-L/H group vs. model group *in vitro* and LC3II, *p* < 0.01 YQF-L/H group vs. model group *in vivo*, *p* < 0.05 YQF-M/H group vs. model group *in vitro*) (**Figure 4** and **Figures 5A–C**). P62 expresses rapidly with the formation and maturation of the autophagosome but decreases with the formation and degradation of autolysosomes. In this study, we found that P62 levels increased in Model groups [χ*^2^* = 16.298 *p* = 0.006, *p* < 0.01 Rapa group vs. model group *in vivo* and *F*(5, 12) = 11.783 p = 0.000, *p* < 0.05 Rapa group vs. model group *in vitro*] but decreased in Rapamycin (*p* < 0.01 *in vivo*, *p* < 0.05 *in vitro*) and YQF groups (*p* < 0.01 YQF-L/M/H group vs. model group *in vivo*, *p* < 0.01 YQF-M/H group vs. model group *in vitro*) (**Figure 4** and **Figures 5A, D**).

### YQF Treatment Suppresses the Activity of mTOR Signaling Pathway

**Figure 6:**
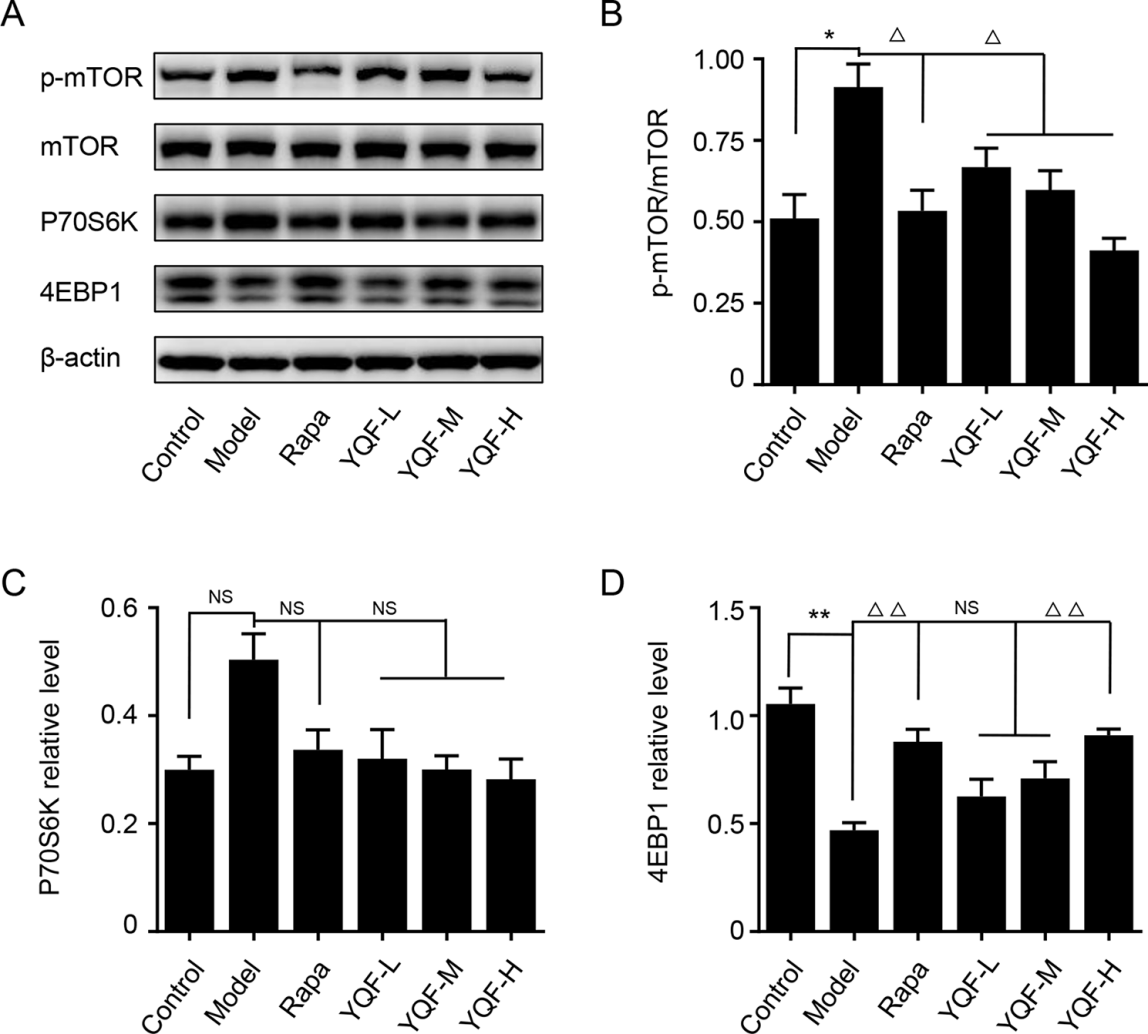
The expression of mTOR, 4EBP1, and P70S6K in APP/PS1 mice intervened with YQF. **(A)** Western blot bands, **(B)** p-mTOR/mTOR, **(C)** P70S6K, **(D)** 4EBP1. Data are expressed as mean ± SEM, n = 3, *p < 0.05 and **p < 0.01 compared with the control group, ^△^*p* < 0.05 and ^△△^*p* < 0.01 compared with the model group, NS, no significant difference.

**Figure 7:**
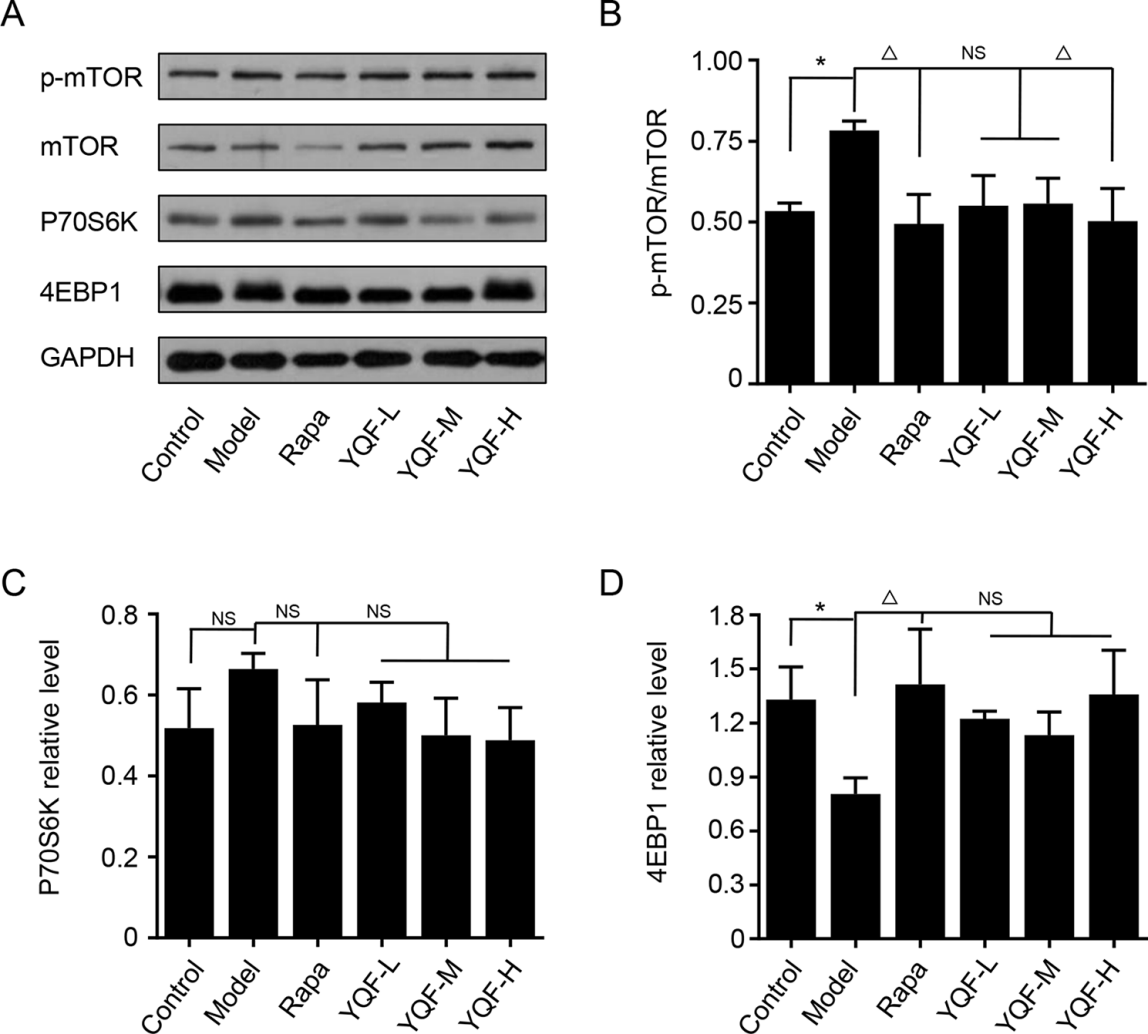
The expression of mTOR, 4EBP1, and P70S6K in HEk293 cells intervened with YQF. **(A)** Western blot bands, **(B)** p-mTOR/mTOR, **(C)** P70S6K, **(D)** 4EBP1. Data are expressed as mean ± SEM, n = 3, *p < 0.05 compared with the control group, ^△^ *p* < 0.05 compared with the model group, NS, no significant difference.

Autophagy is regulated by the mTOR signaling pathway, so we studied how it is affected by YQF. Our results showed that YQF could significantly suppress the activity of this pathway (**Figure 6** and **Figure 7**). We considered p-mTOR/mTOR ratio as the indicator of mTOR activity, and we found significantly elevated mTOR activity in model mice and cells [*F*(5,24) = 7.950 *p* = 0.000, *p* < 0.05 model group vs. control group *in vivo* and *F*(5,12) = 1.990 *p* = 0.153, *p* < 0.05 model group vs. control group *in vitro*]. Rapamycin and YQF could reverse this condition (*p* < 0.05 Rapa group vs. model group *in vivo* and *in vitro*, *p* < 0.05 YQF-H group vs. model group *in vivo* and *in vitro*), in effect suppressing the activity of mTOR. P70S6K and 4EBP1 are two major downstream signaling molecules of mTOR; with the activation of mTOR, P70S6K is activated and 4EBP1 is suppressed. Here, we found that in each group, P70S6K varied directly with pmTOR/mTOR ratio and had an indirect relationship with 4EBP1 levels. However, we did not find significant differences in P70S6K levels between the different study groups. Although rapamycin could significantly promote the expression of 4EBP1, we did not find any difference between YQF groups and the Model group.

### YQF Treatment Promotes the Degradation of Autolysosome *In Vitro*

**Figure 8:**
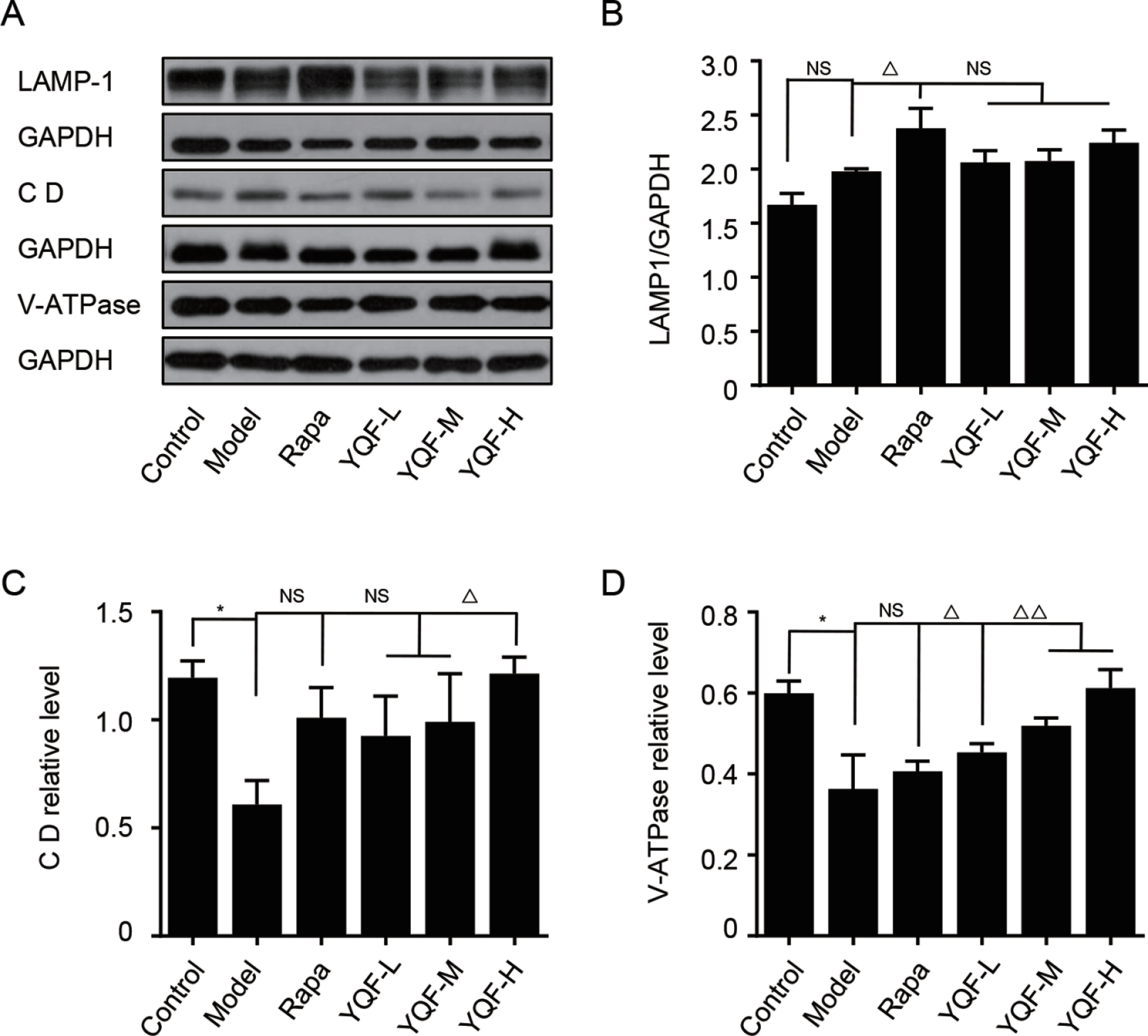
The expression of LAMP-1, Cathepsin D CD and V-ATPase in HEK293 cells intervened with YQF. **(A)** Western blot bands, **(B)** LAMP-1, **(C)** Cathepsin D, **(D)** V-ATPase. Data are expressed as mean ± SEM, n = 3, *p < 0.05 compared with the control group, ^△^p < 0.05 and ^△△^p < 0.01 compared with the model group, NS, no significant difference.

To further explore the effect of YQF on the whole process of autophagy, we analyzed the levels of autolysosome related proteins like lysosomal-associated membrane protein 1 (LAMP-1), cathepsin D, and vacuolar-type H^+^-ATPase (V-ATPase) (**Figure 8**). We found that the levels of LAMP-1 in the Model group and the Control group were not significantly different [*F*(5,12) = 4.257 *p* = 0.019, *p* > 0.05 model group vs. control group]. Rapamycin could promote LAMP-1 expression (*p* < 0.05 Rapa group vs. model group). Although YQF could promote LAMP-1 expression, non-significant differences were found. Cathepsin D and V-ATPase were found to be significantly decreased in the Model group compared with the Control group [*F*(5,12) = 4.322 *p* = 0.018, *p* < 0.05 model group vs. control group and *F*(5,12) = 2.237 *p* = 0.113, *p* < 0.05 model group vs. control group]. Rapamycin could increase the cathepsin D and V-ATPase levels; however, the differences were non-significant. YQF could significantly enhance the expression of cathepsin D (*p* < 0.05 YQF-H group vs. model group) and V-ATPase (*p* < 0.05 YQF-L group vs. model group and *p* < 0.01 YQF-M/H group vs. model group).

## Discussion

Under physiological conditions, autophagy, by recycling damaged organelle and defective proteins, is crucial for the homeostasis of energy metabolism in neural cells. During normal aging or the progression of AD, autophagy activity gradually declines, attributing to the aggregation of Aβ (Pickford et al., 2008; Uchiyama et al., 2008), which is now widely considered as a prominent feature found in AD brain. The impairment of autophagy in AD has been substantiated by the accumulation of autophagosomes in dystrophic neurites (Tanida et al., 2005). Consistent with these results, activation of the autophagy pathway by rapamycin retards Aβ and Tau pathology and enhances cognitive functions (Caccamo et al., 2010; Majumder et al., 2011). Apart from these data, previous study also reported that autophagy participates in the extracellular Aβ secretion, thereby directly affecting Aβ plaque formation (Nilsson et al., 2013). Therefore, considerable efforts have been focusing on autophagy to discover potential targets for AD therapeutic interventions (Zhu et al., 2013; Jiang et al., 2014). Beclin 1, the mammalian orthologue of the yeast Vps30/Apg6 gene, functioning as a scaffold, is necessary for the formation of autophagosome. Previous study showed a reduction of Beclin 1 level in the entorhinal cortex of AD patients (Pickford et al., 2008). In APP transgenic mice, Beclin 1 deficiency exacerbates Aβ deposition and promotes synaptodendritic degeneration. On the contrary, Beclin 1 overexpression reduces amyloid pathology (Pickford et al., 2008). Our results suggest a significant reduction of Beclin 1, accompanied with an increased Aβ accumulation in the brain of *APP/PS1* mice and *APP/PS1* HEK293 cells. Furthermore, results demonstrate that YQF or rapamycin treatment elevates the level of Beclin 1 and reduces the formation of Aβ and amyloid plaques.

Microtubule-associated protein 1 light chain 3 (LC3 I) plays a key role in autophagosome biogenesis/maturation. During autophagy, a cytosolic form of LC3 (LC3 I) is converted from a nonlipidated form (LC3 I) to a phosphatidylethanolamine (PE)-conjugated form (LC3 II), which is recruited to autophagosomal membranes. LC3 II specifically localizes in autophagosome membranes, and it is therefore considered a specific autophagy marker (Lai and Devenish, 2012). Sequestosome-1 (SQSTM1)/p62 is a ubiquitin-LC3-binding protein and also an adaptor protein for the autophagic degradation of misfolded proteins or organelles. p62 itself is a selective autophagy substrate targeted by the autophagy–lysosome degradation system. Hence, the level of p62 inversely correlates with autophagy activity and is considered as an important biomarker for autophagy degradation (Tanida et al., 2005). In accordance with previous data (Lee et al., 2014; Guo et al., 2015), here we show that an impaired autophagic flux is present, as indicated by an increase of LC3 II/LC3 I and p62 level *in vivo* in model mice, whereas with the *in vitro* study, LC3 II/LC3 I decreased but the level of p62 increased in *APP/PS1* double transgenic HEK293 cells, indicating that both the formation and degradation of autophagosome are obstructed in AD. Importantly, in this study, treatment with different doses of YQF rectified the aberrant autophagy activity. Our results show that YQF treatment increases the LC3 II/LC3 I ratio and reduces p62 levels.

In order to explore the underlying mechanism, we evaluated the phosphorylation of mTOR and the lysosomal function. mTOR is a critical negative regulator of autophagy, which senses and integrates signals under various conditions including growth factors, amino acids, hypoxia, and energy levels (Liang, 2010; Kim and Guan, 2015). In the brain, it is primarily expressed in neurons and is involved in synaptic plasticity and cognition (Talboom et al., 2015). In AD, there is ample evidence suggesting that hyperactive mTOR contributes to the accumulation of Aβ and cognitive impairment. mTOR inhibitors reduce AD hallmarks and protect cognitive function (Godoy et al., 2014; Tramutola et al., 2017). In this study, we found that 9-month-old *APP/PS1* mice and *APP/PS1* double transgenic HEK293 cells exhibit hyperphosphorylated mTOR compared with age-matched wild-type control C57BL/6J mice and non-transfected HEK293 cells, which is in accordance with previous study (Zhou et al., 2008). Our study found that YQF and rapamycin treatments retard the abnormal phosphorylation, and thus inhibit the activity of mTOR and promoted autophagy. By phosphorylating the downstream signaling molecules, mTOR activates P70S6K and inactivates 4EBP1, thereby exerting intracellular signal transduction (Perluigi et al., 2015). Although we did not find significant changes in P70S6K activity in each group, we found that the levels of 4EBP1 decreased significantly in Model groups and increased after the *in vivo* YQF treatment, further proof that YQF inhibits the activity of mTOR.

Formation of autolysosomes by the fusion of autophagosome and lysosome is needed for the subsequent completion of substrate clearance. Lysosome function is crucial in this step. Studies found that lysosome dysfunction may be the most important point in the autophagy disorder in AD (Bordi et al., 2016; Ma et al., 2017). LAMP-1 is a lysosome-associated membrane glycoprotein, cathepsin D is an aspartic endo-protease that is ubiquitously distributed in lysosomes, and V-ATPase is an ATP-dependent proton pump (transporting H^+^ ions into the lysosome) that maintains the acidity of lysosomes. Our *in vitro* studies revealed that LAMP-1 levels were largely the same between different groups, but the levels of cathepsin D and V-ATPase decreased significantly in the Model groups, while YQF could increase their levels. This phenomenon indicates that while the levels of lysosomes are not significantly changed in different groups, their functions changed; lysosome function was impaired in Model groups, and YQF could alleviate this defect.

In conclusion, in the present study, we show that long-term administration of YQF ameliorates learning and memory deficits in the *APP/PS1* mice. The presence of Aβ plaques is a key pathological feature of AD. Here, immunohistochemistry results demonstrate that YQF treatment mitigates the formation of amyloid plaques and decreases the levels of Aβ. The results from ELISA assays show that YQF could up-regulate Beclin-1 and LC3 II, down-regulate p62, and inhibit the activity of the mTOR signaling pathway, *in vivo* and *in vitro*. Thus, we conclude that YQF can improve cognitive function and promote Aβ clearance by activating autophagy function *via* suppressing the mTOR pathway, which may provide new insight in the treatment of AD. We have detected several active components of YQF, but we cannot sure which one play the critical role. We will do some more work to research which active components play the key role in YQF.

## Data Availability

The datasets generated for this study are available on request to the corresponding author.

## Ethics Statement

The animal study was reviewed and approved by the animal ethics committee of the Ethics Committee of Xiyuan Hospital, China Academy of Chinese Medical Sciences.

## Author Contributions

YY and ZW contributed equally to this work. YY and ZW performed experiments and data analysis and wrote the manuscript. YC assisted in the *in vivo* experiments and manuscript writing. PL assisted in the *in vitro* experiments. HL and JL supervised the experiments. HL and ML conceived the study and designed the experiments. All the authors reviewed and approved the manuscript.

## Funding

The study was funded by the National Natural Science Foundation of China (No. 81503628, 81573819, and 81873350).

## Conflict of Interest Statement

The authors declare that the research was conducted in the absence of any commercial or financial relationships that could be construed as a potential conflict of interest.

The handling editor declared a shared affiliation, though no other collaboration, with the authors HL, YY, ZW, YC, JL, PL, ML at the time of the review.
