# Supplementary material for: Yizhiqingxin Formula Alleviates Cognitive Deficits and Enhances Autophagy via mTOR Signaling Pathway Modulation in Early Onset Alzheimer’s Disease Mice
Source: Front Pharmacol. 2019 Sep 17;10:1041. doi: 10.3389/fphar.2019.01041 (PMC6758600; doi:10.3389/fphar.2019.01041)
Supplement: Supplementary file 1 [file DataSheet_1.docx]

Yizhiqingxin Formula (YQF) is a traditional Chinese formula composed of *Panax ginseng* (radix), *Coptis chinensis* (rhizome)， and *Conioselinum anthriscoides 'Chuanxiong'* (rhizome). Previously YQF is called Fuzheng Quxie Decoction which originates from Huannao Yicong Formula (HYF). HYF consists of *Radix Polygoni Multiflori Praeparata* (*Polygonum multiflorum* Thunb., *Reynoutria multiflora* (Thunb.) Moldenke), Rhizoma acori tatarinowii (Acorus tatarinowii Schott), and the three contained in YQF.

HYF has been granted a technical invention letter of patent in 2011, by National Intellectual Property Administration (NO. ZL201110007105.6). Clinical evidence showed that HYF alleviated cognitive dysfunction in patients with Mild-to-Moderate Alzheimer’s Disease (Yang et al., 2018). In vivo study revealed that HYF mitigated behavioral alternation in AD rats and decreased the expression of TTBK1, GSK-3 β, and CDK-5 in the hippocampal CA1 region (Cao et al., 2016).

In addition, our previous work found that YQF could ameliorate cognitive deficits and tau hyperphosphorylation in SAMP8 mice (Yang et al., 2017); vascular endothelial growth factor (VEGF) and VEGF receptor were the other molecular targets of YQF (Wang et al., 2018).

References

Yang, Y., Jia, X., Feng, J., Wang, Z., Cao, Y., Liu, J., et al. (2017). Fuzheng Quxie Decoction Ameliorates Learning and Memory Impairment in SAMP8 Mice by Decreasing Tau Hyperphosphorylation. Evid. Based Complement. Alternat. Med. 2017. doi: 10.1155/2017/5934254

Wang, F., Feng, J., Yang, Y., Liu, J., Liu, M., Wang, Z., et al. (2018). The Chinese herbal formula Fuzheng Quxie decoction attenuates cognitive impairment and protects cerebrovascular function in SAMP8 mice. Neuropsychiatr Dis. Treat. 14, 3037-3051. doi:10.2147/ndt. S175484

Yang, Y., Liu, J. P., Fang, J. Y., Wang, H. C., Wei, Y., Cao, Y., et al. (2018). Effect and Safety of Huannao Yicong Formula (还脑益聪方) on Patients with Mild-to-Moderate Alzheimer’s Disease: A Randomized, Double-Blinded, Donepezil-Controlled Trial. Chin J Integr Med. doi: 10.1007/s11655-018-3054-7

Cao, Y., Jia, X., Wei, Y., Liu, M., Liu, J., & Li, H. (2016). Traditional Chinese Medicine Huannao Yicong Decoction Extract Decreases Tau Hyperphosphorylation in the Brain of Alzheimer’s Disease Model Rats Induced by Aβ1–42. Evid. Based Complement. Alternat. Med. 2016. doi: 10.1155/2016/6840432
