# Supplementary material for: Yizhiqingxin Formula Alleviates Cognitive Deficits and Enhances Autophagy via mTOR Signaling Pathway Modulation in Early Onset Alzheimer’s Disease Mice
Source: Front Pharmacol. 2019 Sep 17;10:1041. doi: 10.3389/fphar.2019.01041 (PMC6758600; doi:10.3389/fphar.2019.01041)
Supplement: Supplementary file 2 [file Table_1.docx]

1. Chromatographic conditions for alkaloids in *Rhizoma Coptidis*

A Symmetry Luna C18 chromatographic column (150mm×2.1mm，025331036119 15, Waters, USA) was used, the filter sieve plate with aperture of 0.2µm was used as the pre-column (6/22/2010, ESA, USA), column temperature was 20℃, injection volume was 10μL, automatic sampler maintain at the room temperature, and the running time was 9.5 minutes. The water phase in mobile phase was aqueous solution containing 0.1% formic acid and organic phase was equivalent mixture of methanol and acetonitrile (containing 0.1% formic acid), the velocity of flow was 0.23 mL/min, the gradient elution method was as follow table:

Mobile Phase of *Rhizoma Coptidis* (LC-MS/MS)

|  | Time(min) | Flow (μL/min) | Water(%) | Organic(%) |
| --- | --- | --- | --- | --- |
| 1 | 0.00 | 230 | 80.0 | 20.0 |
| 2 | 2.00 | 230 | 45.0 | 55.0 |
| 3 | 3.00 | 230 | 45.0 | 55.0 |
| 4 | 3.01 | 230 | 80.0 | 20.0 |
| 5 | 9.50 | 230 | 80.0 | 20.0 |

(2) Mass spectrometric conditions for alkaloids in Rhizoma Coptidis

The ion source was electrospray ionization (ESI), curtain gas 15 p.s.i., internal temperature of ion source was 600℃, gas in in source: GS1 (60 p.s.i), GS2 (50p.s.i), ion spray voltage (IS) was 5500 V, collision gas (CAD): High, detection method was positive detection, and the scanning mode was multiple reaction monitoring (MRM), ion pairs for quantitative analysis was shown in the following table:

MRM Parameters

| **#** | Name | Q1 MS (Da) | Q3 MS (Da) | Time  (msec) | DP  (volts) | EP  (volts) | CE  (volts) | CXP  (volts) |
| --- | --- | --- | --- | --- | --- | --- | --- | --- |
| 1 | Ber | 336.2 | 320.2 | 100 | 100 | 10 | 43 | 10 |
| 2 | THP | 356.2 | 192.1 | 100 | 130 | 10 | 38 | 10 |
| 3 | Pal | 352.2 | 336.2 | 100 | 70 | 10 | 40 | 10 |
| 4 | DHC | 366.2 | 350.2 | 100 | 142 | 10 | 40 | 10 |
| 5 | Cor | 370.2 | 192.1 | 100 | 184 | 10 | 44 | 10 |
| 6 | Wor | 334.1 | 306.2 | 100 | 120 | 10 | 42 | 10 |
| 7 | Pro | 354.1 | 188.1 | 100 | 141 | 10 | 45 | 10 |
| 8 | eBer | 336.2 | 320.1 | 100 | 100 | 10 | 44 | 10 |
| 9 | Cop | 320.1 | 292.1 | 100 | 100 | 10 | 41 | 10 |
| 10 | ACrp | 370 | 188.1 | 100 | 90 | 10 | 35 | 10 |
| 11 | Gla | 356.2 | 325.2 | 100 | 70 | 10 | 20 | 10 |
| 12 | Jat | 338.1 | 322.2 | 100 | 100 | 10 | 40 | 10 |
| 13 | THJ | 342.2 | 178.1 | 100 | 100 | 10 | 40 | 10 |
| 14 | THB | 340.2 | 176.1 | 100 | 70 | 10 | 39 | 10 |

(3) Chromatographic conditions for ginsenosides in *Radix Ginseng*

A Symmetry Luna C18 chromatographic column (150mm×2.1mm，025331036119 15, Waters, USA) was used, the filter sieve plate with aperture of 0.2µm was used as the pre-column (6/22/2010, ESA, USA), column temperature was 10℃, injection volume was 10μL, the temperature of the automatic sampler was 4℃, and the running time was15.0 minutes. The water phase in mobile phase was aqueous solution containing 0.01% formic acid (containing 10% organic phase) and the organic phase was equivalent mixture of methanol and acetonitrile (containing 0.01% formic acid), the velocity of flow was 0.23 mL/min, the gradient elution method was as follow table:

Mobile Phase of Ginseng (LC-MS/MS)

|  | Time(min) | Flow (μL/min) | Water(%) | Organic(%) |
| --- | --- | --- | --- | --- |
| 1 | 0.00 | 230 | 70.0 | 30.0 |
| 2 | 0.20 | 230 | 70.0 | 30.0 |
| 3 | 2.00 | 230 | 28.0 | 72.0 |
| 4 | 6.50 | 230 | 28.0 | 72.0 |
| 5 | 6.51 | 230 | 70.0 | 30.0 |

(4) Mass spectrometric conditions for ginsenosides in *Radix Ginseng*

The ion source was electrospray ionization (ESI), curtain gas 10 p.s.i., internal temperature of ion source was 600℃, gas in in source: GS1 (50 p.s.i), GS2 (50p.s.i), ion spray voltage (IS) was 6500 V, collision gas (CAD): High, detection method was positive detection, and the scanning mode was multiple reaction monitoring (MRM), ion pairs for quantitative analysis was shown in the following table:

MRM Parameters

| **#** | Name | Q1 MS (Da) | Q3 MS (Da) | Time  (msec) | DP  (volts) | EP  (volts) | CE  (volts) | CXP  (volts) |
| --- | --- | --- | --- | --- | --- | --- | --- | --- |
| 1 | Rg_1_ | 823.6 | 643.6 | 80 | 190 | 10 | 53 | 15 |
| 2 | Re | 969.7 | 789.7 | 80 | 200 | 10 | 63 | 17 |
| 3 | Rb_1_ | 1131.8 | 789.5 | 80 | 240 | 10 | 80 | 16 |
| 4 | Rd | 969.7 | 789.7 | 80 | 200 | 10 | 70 | 16 |
| 5 | Rb_2/3_ | 1101.7 | 335.3 | 80 | 210 | 10 | 80 | 15 |
| 6 | Rc | 1101.7 | 335.3 | 80 | 210 | 10 | 80 | 15 |
| 7 | Rf | 823.6 | 365.3 | 80 | 185 | 10 | 72 | 10 |
| 8 | F1 | 661.4 | 203.2 | 80 | 130 | 10 | 50 | 11 |
| 9 | Rg_2_ | 807.6 | 349.2 | 80 | 194 | 10 | 63 | 10 |
| 10 | F11 | 823.7 | 497.5 | 80 | 210 | 10 | 72 | 10 |
| 11 | Rh_1_ | 661.6 | 481.2 | 80 | 160 | 10 | 52 | 10 |

(5) Chromatographic conditions for active components in *Rhizoma Chuanxiong*

A Kinetex C18 chromatographic column (Phenomenex, USA) was used, the filter sieve plate with aperture of 0.2µm was used as the pre-column (ESA, USA), column temperature was 10℃, injection volume was 10μL, the temperature of the automatic sampler was 10℃, and the running time was15.0 minutes. The water phase in mobile phase was aqueous solution containing 0.05% formic acid (containing 10% organic phase) and the organic phase was equivalent mixture of methanol and acetonitrile (containing 0.05% formic acid), the velocity of flow was 0.23 mL/min, the gradient elution method was as follow table:

Mobile Phase of Tianlong NEG (LC-MS/MS)

|  | Time(min) | Flow (μL/min) | Water(%) | Organic(%) |
| --- | --- | --- | --- | --- |
| 1 | 0.00 | 300 | 95.0 | 5.0 |
| 2 | 0.50 | 300 | 95.0 | 5.0 |
| 3 | 1.50 | 300 | 35.0 | 65.0 |
| 4 | 4.50 | 300 | 35.0 | 65.0 |
| 5 | 5.50 | 300 | 95.0 | 5.0 |
| 6 | 6.51 | 300 | 95.0 | 5.0 |

(6) Mass spectrometric conditions for active components in *Rhizoma Chuanxiong*

The ion source was electrospray ionization (ESI), curtain gas 20 p.s.i., internal temperature of ion source was 600℃, gas in in source: GS1 (55 p.s.i), GS2 (55p.s.i), ion spray voltage (IS) was 4500 V, collision gas (CAD): Medium, detection method was nevatige detection, and the scanning mode was multiple reaction monitoring (MRM), ion pairs for quantitative analysis was shown in the following table:

MRM Parameters

| **#** | Name | Q1 MS (Da) | Q3 MS (Da) | Time  (msec) | DP  (volts) | EP  (volts) | CE  (volts) | CXP  (volts) |
| --- | --- | --- | --- | --- | --- | --- | --- | --- |
| 1 | Ferulic Acid | 193.0 | 133.9 | 80 | -40 | -10 | -21 | -5 |
| 2 | IS | 149.9 | 106.9 | 80 | -60 | -10 | -25 | -5 |
